# Risk of all-cause mortality according to the European Society of Cardiology risk categories in individuals with type 2 diabetes: the Renal Insufficiency And Cardiovascular Events (RIACE) Italian Multicenter Study

**DOI:** 10.1007/s00592-022-01942-8

**Published:** 2022-07-28

**Authors:** Emanuela Orsi, Anna Solini, Enzo Bonora, Martina Vitale, Monia Garofolo, Cecilia Fondelli, Roberto Trevisan, Monica Vedovato, Franco Cavalot, Luigi Laviola, Susanna Morano, Giuseppe Pugliese, for the Renal Insufficiency And Cardiovascular Events (RIACE) Study Group

**Affiliations:** 1 Diabetes Unit, IRCCS “Cà Granda - Ospedale Maggiore Policlinico” Foundation, Milan, Italy; 2 Department of Surgical, Medical, Molecular and Critical Area Pathology, University of Pisa, Pisa, Italy; 3 Division of Endocrinology, Diabetes and Metabolism, University and Hospital Trust of Verona, Verona, Italy; 4 Department of Clinical and Molecular Medicine, “La Sapienza” University, Via di Grottarossa, 1035-1039, 00189 Rome, Italy; 5 Department of Clinical and Experimental Medicine, University of Pisa, Pisa, Italy; 6 Diabetes Unit, University of Siena, Siena, Italy; 7 Endocrinology and Diabetes Unit, Azienda Ospedaliera Papa Giovanni XXIII, Bergamo, Italy; 8 Department of Clinical and Experimental Medicine, University of Padua, Padua, Italy; 9 Department of Clinical and Biological Sciences, University of Turin, Orbassano, Italy; 10 Department of Emergency and Transplants, Section of Internal Medicine, Endocrinology, Andrology and Metabolic Diseases, University of Bari Aldo Moro, Bari, Italy; 11 Department of Experimental Medicine, “La Sapienza” University, Rome, Italy

**Keywords:** Type 2 diabetes, All-cause mortality, Atherosclerotic cardiovascular disease, Target organ damage, Atherosclerotic cardiovascular disease risk factors

## Abstract

**Aims:**

The 2019 and 2021 European Society of Cardiology (ESC) classifications stratified patients with type 2 diabetes into three categories according to the 10-year risk of death from atherosclerotic cardiovascular disease (ASCVD). The very high-risk category included individuals with established ASCVD, target organ damage (TOD), and/or, in the 2019 classification only, ≥ 3 additional ASCVD risk factors. We assessed risk of all-cause mortality according to the two ESC classifications in the Renal Insufficiency And Cardiovascular Events cohort.

**Methods:**

Participants (n = 15,773) were stratified based on the presence of ASCVD, TOD, and ASCVD risk factors at baseline (2006–2008). Vital status was retrieved in 2015.

**Results:**

Less than 1% of participants fell in the moderate-risk category. According to the 2019 classification, ~ 1/3 fell in the high-risk and ~ 2/3 in the very high-risk category, whereas the opposite occurred with the 2021 classification. Mortality risk increased across categories according to both classifications. Among very high-risk patients, mortality was much lower in those with ≥ 3 additional ASCVD risk factors and almost equal in those with TOD and ASCVD ± TOD, using the 2019 classification, whereas it was much higher in those with ASCVD + TOD and, to a lesser extent, TOD only than in those with ASCVD only, using the 2021 classification.

**Conclusions:**

The negligible number of moderate-risk patients suggests that these classifications might overestimate risk of ASCVD death. Downgrading patients with ≥ 3 additional ASCVD risk factors to the high-risk category is consistent with mortality data. Risk of death is very high in the presence of TOD irrespective of established ASCVD.

*Trial registration*: ClinicalTrials.gov, NCT00715481.

**Supplementary Information:**

The online version contains supplementary material available at 10.1007/s00592-022-01942-8.

## Introduction

Mortality is approximately twice higher in diabetic than in non-diabetic individuals [1, 2], mainly but not exclusively due to an excess risk of atherosclerotic cardiovascular disease (ASCVD) [3]. However, death rates have consistently decreased over time in people with diabetes [4], mainly due to reduction in ASCVD events such as myocardial infarction and stroke [5]. This is likely related to improved treatment of multiple ASCVD risk factors, including hyperglycemia, dyslipidemia, and hypertension [6]. However, the burden from ASCVD remains disproportionately high in diabetic patients, especially in those with type 2 diabetes, thus requiring a systematic estimation of ASCVD risk in order to initiate therapeutic strategies for risk reduction [7].

Several prediction tools have been developed to estimate the risk of ASCVD events and all-cause or ASCVD mortality; however, these algorithms, which are mainly based on ASCVD risk factors, have numerous limitations resulting in insufficient performance, especially in people with type 2 diabetes [8]. Thus, at variance with guidelines of other scientific societies [9, 10], those of the European Society of Cardiology (ESC) [11, 12] recommend the use of a newly-developed, non-validated risk stratification system based on the presence of ASCVD risk factors, target organ damage (TOD), and/or previous ASCVD event(s) instead of one of the existing ASCVD risk prediction tools. Specifically, the 2019 ESC guidelines [11] identified three categories according to the 10-year risk of death from ASCVD, i.e., moderate (< 5% risk), high (5–10% risk), and very high (> 10% risk). The highest risk category included patients with established ASCVD, those with severe TOD (including microangiopathy and left ventricular hypertrophy), and/or those with at least three additional ASCVD risk factors. In 2021, this classification has been revised, mainly by restricting the definition of very high-risk individuals to patients with ASCVD and/or severe TOD, which included only a broader definition of microvascular disease [12].

Here, we analyzed the large cohort of patients with type 2 diabetes from the Renal Insufficiency And Cardiovascular Events (RIACE) Italian Multicenter Study to assess the distribution of a real-world sample into the 2019 and 2021 ESC risk categories and the risk of all-cause mortality in each of these categories.

## Methods

### Design

The RIACE Italian Multicenter Study is an observational, prospective, cohort study on the impact of estimated glomerular filtration rate (eGFR) on morbidity and mortality in people with type 2 diabetes [13].

### Patients

The study population included 15,773 Caucasian patients (after excluding 160 individuals with missing or implausible values), consecutively attending 19 hospital-based, tertiary referral outpatient Diabetes Clinics of the National Health Service throughout Italy in the years 2006–2008. Exclusion criteria were dialysis or renal transplantation [13].

### All-cause mortality

The vital status of study participants on 31 October 2015 was verified by interrogating the Italian Health Card database (http://sistemats1.sanita.finanze.it/wps/portal/), which provides updated and reliable information on all current Italian residents [14].

### Baseline measurements

Baseline data were collected using a standardized protocol across participating centers [13].

Participants underwent a structured interview in order to collect the following information: age at the time of the interview, smoking status (never, former, current), known diabetes duration, severe co-morbidities (including chronic obstructive pulmonary disease, chronic liver disease, and cancer), and current glucose-, lipid-, and blood pressure (BP)-lowering treatments.

Body mass index (BMI) was calculated from weight and height, whereas waist circumference was estimated from log-transformed BMI values; BP was measured with a sphygmomanometer with the patients seated with the arm at the heart level.

Hemoglobin A_1c_ (HbA_1c_) was measured by HPLC using DCCT-aligned methods; triglycerides and total and HDL cholesterol were determined in fasting blood samples by colorimetric enzymatic methods; LDL cholesterol concentration was estimated using the Friedewald formula.

The presence of diabetic kidney disease (DKD) was assessed by measuring albuminuria and serum creatinine, as previously detailed [13, 15]. Albumin excretion rate was obtained from 24-h urine collections or calculated from albumin-to-creatinine ratio in early morning, first voided urine samples; albumin concentration in urines was measured by immunonephelometry or immunoturbidimetry, in the absence of interfering clinical conditions. Serum (and urine) creatinine was measured by the modified Jaffe method, traceable to IDMS, and eGFR was calculated by the Chronic Kidney Disease Epidemiology Collaboration equation. Patients were then assigned to one of the following DKD phenotypes: no DKD, albuminuria alone (albuminuric DKD with preserved eGFR), reduced eGFR alone (non-albuminuric DKD), or both albuminuria and reduced eGFR (albuminuric DKD with reduced eGFR), as previously reported [16].

The presence of diabetic retinopathy (DR) was assessed in each center by an expert ophthalmologist by dilated fundoscopy [17]. Patients with mild or moderate non-proliferative DR were classified as having non-advanced DR, whereas those with severe non-proliferative DR, proliferative DR, or maculopathy were grouped into the advanced, sight-threatening DR category. DR grade was assigned based on the worse eye.

Previous major acute ASCVD events, including myocardial infarction; stroke; foot ulcer/gangrene/amputation; and coronary, carotid, and lower limb revascularization, were adjudicated based on hospital discharge records by an ad hoc committee in each center [18].

### Statistical analysis

For the purpose of the current analysis, the RIACE participants were classified according to the 2019 or the 2021 ESC risk categories, with minor modifications due to the lack of information on left ventricular hypertrophy and neuropathy, respectively (Supplemental Table 1).

Data are expressed as mean ± SD for continuous variables, and number of cases and percentage for categorical variables. Comparisons among categories were performed by one-way ANOVA for continuous variables and Pearson’s Chi-square test for categorical variables.

Crude mortality rates were described as events per 1000 patient-years, with 95% exact Poisson confidence intervals (CIs) and adjusted for age and sex by a Poisson regression model. Kaplan–Meier survival probabilities for all-cause mortality were estimated according to the 2019 or 2021 risk categories, and differences were analyzed using the log-rank statistic. The hazard ratios (HRs) and their 95% CIs were estimated by Cox proportional hazards regression, using the moderate risk category as reference. These analyses were sequentially adjusted for (a) parameters that were not used for patients’ categorization, i.e., sex and severe comorbidities, including chronic obstructive pulmonary disease, chronic liver disease, and cancer (model 1); (b) parameters that were used only for defining the moderate risk category in the 2021 classification, i.e., glycemic control (HbA_1c_ and anti-hyperglycemic treatment) (model 2); and (c) additional ASCVD risk factors that were used for patients’ categorization, i.e., age, smoking, diabetes duration, BMI, triglycerides, total and HDL cholesterol, systolic and diastolic BP, and lipid-lowering and anti-hypertensive treatment (model 3).

All *p* values were two-sided, and a *p* < 0.05 was considered statistically significant. Statistical analyses were performed using SPSS version 13.0 (SPSS Inc., Chicago, IL, USA).

## Results

The distribution and clinical features of the RIACE participants according to the 2019 and 2021 ESC risk categories and subcategories are shown in Tables 1 and 2, respectively.

**Table 1:** Baseline clinical features of study participants by 2019 ESC risk categories and subcategories

Variables	Moderate risk	High risk	Very high risk	P*	TOD	CVD	TOD + ASCVD	*P*^†^
*N* (%)	60 (0.4)	5612 (35.8)	9984 (63.8)	NA	4978 (31.8)	1386 (8.9)	3620 (23.1)	NA
Deaths, *n* (%)	3 (5.0)	710 (12.7)	2889 (28.9)	< 0.0001	1072 (21.5)	448 (32.3)	1369 (37.8)	< 0.0001
Age, years	41.3 ± 7.1	62.6 ± 9.5	69.0 ± 9.9	< 0.0001	68.9 ± 10.3	67.5 ± 9.7	69.7 ± 9.3	< 0.0001
Age > 70 years, *n*%	0 (0.0)	765 (13.6)	4740 (47.5)	< 0.0001	2554 (51.3)	512 (36.9)	1674 (46.2)	< 0.0001
Sex, *n* (%)				< 0.0001				< 0.0001
Females	25 (41.7)	2295 (40.9)	4434 (44.4)		2654 (53.3)	607 (43.8)	1173 (32.4)	
Males	35 (58.3)	3317 (59.1)	5550 (55.6)		2324 (46.7)	779 (56.2)	2447 (67.6)	
Smoking, *n* (%)				< 0.0001				< 0.0001
Never	51 (85.0)	3523 (62.8)	5275 (52.8)		2706 (54.4)	832 (60.0)	1737 (48.0)	
Former	9 (15.0)	1566 (27.9)	2832 (28.4)		1110 (22.3)	344 (24.8)	1378 (38.1)	
Current	0 (0.0)	523 (9.3)	1877 (18.8)		1162 (23.3)	210 (15.2)	505 (14.0)	
Diabetes duration, years	3.7 ± 2.6	10.7 ± 9.0	14.6 ± 10.5	< 0.0001	12.7 ± 10.1	17.1 ± 10.3	16.4 ± 10.6	< 0.0001
Diabetes duration > 10 years, *n* (%)	0 (0.0)	2251 (40.1)	5620 (56.3)	< 0.0001	2332 (46.8)	965 (69.6)	2323 (64.2)	< 0.0001
HbA_1c_, %	7.45 ± 1.92	7.34 ± 1.41	7.66 ± 1.54	< 0.0001	7.53 ± 1.48	7.96 ± 1.67	7.73 ± 1.54	< 0.0001
(mmol mol^−1^)	(57.9 ± 21.0)	(56.7 ± 15.4)	(60.2 ± 16.8)		(58.8 ± 16.2)	(63.5 ± 18.3)	(61.0 ± 16.8)	
BMI, kg m^−2^	25.2 ± 2.8	27.2 ± 4.4	30.0 ± 5.3	< 0.0001	31.0 ± 5.4	29.4 ± 5.2	28.8 ± 4.8	< 0.0001
Obesity, *n* (%)	0 (0.0)	826 (14.7)	4850 (48.6)	< 0.0001	2994 (60.1)	553 (39.9)	1303 (36.0)	< 0.0001
Waist circumference, cm	94.8 ± 5.9	98.9 ± 8.9	104.6 ± 10.6	< 0.0001	106.4 ± 10.8	103.2 ± 10.5	102.5 ± 10.0	< 0.0001
Triglycerides, mmol l^−1^	1.03 ± 0.58	1.46 ± 0.94	1.64 ± 1.02	< 0.0001	1.64 ± 1.00	1.71 ± 1.11	1.62 ± 1.02	< 0.0001
Total cholesterol, mmol l^−1^	4.05 ± 0.59	4.75 ± 0.97	4.81 ± 1.00	< 0.0001	4.99 ± 0.95	4.87 ± 1.04	4.53 ± 0.99	< 0.0001
HDL cholesterol, mmol l^−1^	1.40 ± 0.39	1.33 ± 0.36	1.27 ± 0.35	< 0.0001	1.30 ± 0.35	1.28 ± 0.35	1.23 ± 0.34	
Non-HDL cholesterol, mmol l^−1^	2.65 ± 0.46	3.43 ± 0.93	3.54 ± 0.96	< 0.0001	3.69 ± 0.93	3.59 ± 1.01	3.30 ± 0.94	< 0.0001
LDL cholesterol, mmol l^−1^	2.18 ± 0.36	2.77 ± 0.84	2.80 ± 0.85	< 0.0001	2.96 ± 0.83	2.81 ± 0.85	2.58 ± 0.83	< 0.0001
Dyslipidemia, n (%)	0 (0.0)	3893 (69.4)	8963 (89.8)	< 0.0001	4699 (94.4)	1124 (81.1)	3140 (86.7)	< 0.0001
Systolic BP, mmHg	120.4 ± 11.5	134.1 ± 16.3	140.4 ± 18.5	< 0.0001	141.6 ± 17.8	142.2 ± 19.1	138.0 ± 19.0	< 0.0001
Diastolic BP, mmHg	76.7 ± 9.6	78.4 ± 8.8	79.0 ± 9.7	< 0.0001	80.0 ± 9.6	79.2 ± 10.2	77.5 ± 9.6	< 0.0001
Pulse pressure, mmHg	43.7 ± 8.8	55.7 ± 14.1	61.4 ± 16.2	< 0.0001	61.7 ± 15.8	63.1 ± 16.6	60.5 ± 16.4	< 0.0001
Hypertension, *n* (%)	0 (0.0)	2973 (53.0)	9238 (92.5)	< 0.0001	4749 (95.4)	1202 (86.7)	3287 (90.8)	< 0.0001
Anti-hyperglycemic treatment, *n* (%)				< 0.0001				< 0.0001
Lifestyle	13 (21.7)	1052 (18.7)	1048 (10.5)		697 (14.0)	59 (4.3)	292 (8.1)	
Non-insulin	24 (40.0)	3524 (62.8)	6071 (60.8)		3359 (67.5)	674 (48.6)	2038 (56.3)	
Insulin	23 (38.3)	1036 (18.5)	2865 (28.7)		922 (18.5)	653 (47.1)	1290 (35.6)	
Lipid-lowering treatment, *n* (%)	0 (0.0)	1754 (31.3)	5484 (54.9)	< 0.0001	2467 (49.6)	608 (43.9)	2409 (66.5)	< 0.0001
Anti-hypertensive treatment, *n* (%)	0 (0.0)	2569 (45.8)	8503 (85.2)	< 0.0001	4260 (85.6)	1090 (78.6)	3153 (87.1)	< 0.0001
Anti-platelet treatment, *n* (%)	3 (5.0)	1222 (21.8)	5023 (50.3)	< 0.0001	1858 (37.3)	590 (42.6)	2575 (71.1)	< 0.0001
Anti-coagulant treatment, *n* (%)	0 (0.0)	113 (2.0)	556 (5.6)	< 0.0001	191 (3.8)	48 (3.5)	317 (8.8)	< 0.0001
Albuminuria, mg day^−1^	10.9 ± 10.8	20.1 ± 32.2	102.0 ± 393.1	< 0.0001	29.9 ± 45.5	302.8 ± 643.8	124.3 ± 491.7	< 0.0001
Serum creatinine, μmol l^−1^	66.1 ± 15.1	73.3 ± 19.8	85.6 ± 39.8	< 0.0001	76.6 ± 21.8	100.6 ± 71.7	92.3 ± 38.9	< 0.0001
eGFR, ml min^−1^·1.73 m^−2^	108.8 ± 13.8	87.8 ± 17.1	75.8 ± 21.6	< 0.0001	79.6 ± 19.0	71.5 ± 26.4	72.3 ± 22.0	< 0.0001
DKD phenotype, *n* (%)				< 0.0001				< 0.0001
No DKD	55 (91.7)	4485 (79.9)	5444 (54.5)		3199 (64.3)	475 (34.3)	1770 (48.9)	
Albuminuric DKD with preserved eGFR	5 (8.3)	736 (13.1)	2225 (22.3)		956 (19.2)	476 (34.3)	793 (21.9)	
Nonalbuminuric DKD	0 (0.0)	267 (4.8)	1209 (12.1)		582 (11.7)	132 (9.5)	495 (13.7)	
Albuminuric DKD with reduced eGFR	0 (0.0)	124 (2.2)	1106 (11.1)		241 (4.8)	303 (21.9)	562 (15.5)	
DR, *n* (%)				0.001				< 0.0001
No DR	59 (98.3)	5019 (89.4)	7111 (71.2)		4323 (86.8)	318 (22.9)	2470 (68.2)	
Non-advanced DR	1 (1.7)	593 (10.6)	1353 (13.6)		655 (13.2)	78 (5.6)	620 (17.1)	
Advanced DR	0 (0.0)	0 (0.0)	1520 (15.2)		0 (0.0)	990 (71.4)	530 (14.6)	
TOD, *n* (%)	0 (0.0)	0 (0.0)	2184 (21.9)	< 0.0001	0 (0.0)	1386 (100.0)	798 (22.0)	< 0.0001
ASCVD, *n* (%)								
Any	0 (0.0)	0 (0.0)	3620 (36.3)	< 0.0001	0 (0.0)	0 (0.0)	3620 (100.0)	< 0.0001
Myocardial infarction	0 (0.0)	0 (0.0)	1742 (17.4)	< 0.0001	0 (0.0)	0 (0.0)	1742 (48.1)	< 0.0001
Coronary revascularization	0 (0.0)	0 (0.0)	1579 (15.8)	< 0.0001	0 (0.0)	0 (0.0)	1579 (43.6)	< 0.0001
Any coronary event	0 (0.0)	0 (0.0)	2396 (24.0)	< 0.0001	0 (0.0)	0 (0.0)	2396 (66.2)	< 0.0001
Stroke	0 (0.0)	0 (0.0)	513 (5.1)	< 0.0001	0 (0.0)	0 (0.0)	513 (14.2)	< 0.0001
Carotid revascularization	0 (0.0)	0 (0.0)	856 (8.6)	< 0.0001	0 (0.0)	0 (0.0)	856 (23.6)	< 0.0001
Any carotid event	0 (0.0)	0 (0.0)	1292 (12.9)	< 0.0001	0 (0.0)	0 (0.0)	1292 (35.7)	< 0.0001
Ulcer/gangrene/amputation	0 (0.0)	0 (0.0)	556 (5.6)	< 0.0001	0 (0.0)	0 (0.0)	556 (15.4)	< 0.0001
Lower limb revascularization	0 (0.0)	0 (0.0)	450 (4.5)	< 0.0001	0 (0.0)	0 (0.0)	450 (12.4)	< 0.0001
Any peripheral event	0 (0.0)	0 (0.0)	883 (8.8)	< 0.0001	0 (0.0)	0 (0.0)	883 (24.4)	< 0.0001
Comorbidities *n* (%)								
Any	8 (13.3)	933 (16.6)	1846 (18.5)	0.009	839 (16.9)	237 (17.1)	770 (21.3)	< 0.0001
COPD	1 (1.7)	142 (2.5)	531 (5.3)	< 0.0001	244 (4.9)	70 (5.1)	217 (6.0)	< 0.0001
Chronic liver disease	6 (10.0)	526 (9.4)	829 (8.3)	0.070	331 (6.6)	104 (7.5)	394 (10.9)	< 0.0001
Cancer	3 (5.0)	348 (6.2)	680 (6.8)	0.298	355 (7.1)	85 (6.1)	240 (6.6)	0.345

*P* value versus moderate risk of 3-group * and 3-group^†^ comparisons. *ESC* European Society of Cardiology, *HbA*_*1c*_ hemoglobin A_1c_, *BMI* body mass index, *BP* blood pressure, *eGFR* estimated glomerular filtration rate, *DKD* diabetic kidney disease, *DR* diabetic retinopathy, *TOD* target organ damage, *ASCVD* atherosclerotic cardiovascular disease, *COPD* chronic obstructive pulmonary disease

**Table 2:** Baseline clinical features of study participants by 2021 ESC risk categories and subcategories

Variables	Moderate risk	High risk	Very high risk	P*	TOD	CVD	TOD + ASCVD	*P*^†^
*N* (%)	126 (0.8)	10,427 (66.6)	5103 (32.6)	NA	1483 (9.5)	2608 (16.7)	1012 (6.5)	NA
Deaths, *n* (%)	7 (5.6)	1639 (15.7)	1956 (38.3)	< 0.0001	587 (39.6)	779 (29.9)	590 (58.3)	< 0.0001
Age, years	55.6 ± 10.6	65.1 ± 10.3	69.9 ± 9.5	< 0.0001	70.4 ± 10.0	68.7 ± 9.1	72.3 ± 9.4	< 0.0001
Age > 70 years, *n*%	0 (0.0)	3088 (29.6)	2417 (47.4)	< 0.0001	743 (50.1)	1077 (41.3)	597 (59.0)	< 0.0001
Sex, *n* (%)				< 0.0001				< 0.0001
Females	39 (31.0)	4879 (46.8)	1836 (36.0)		663 (44.7)	830 (31.8)	343 (33.9)	
Males	87 (69.0)	5548 (53.2)	3267 (64.0)		820 (55.3)	1778 (68.2)	669 (66.1)	
Smoking, *n* (%)				< 0.0001				< 0.0001
Never	93 (73.8)	6154 (59.0)	2602 (51.0)		865 (58.3)	1242 (47.6)	495 (48.9)	
Former	33 (26.2)	2585 (24.8)	1789 (35.1)		411 (27.7)	990 (38.0)	388 (38.3)	
Current	0 (0.0)	1688 (16.2)	712 (14.0)		207 (14.0)	376 (14.4)	129 (12.7)	
Diabetes duration, years	4.7 ± 2.9	11.7 ± 9.6	16.4 ± 10.6	< 0.0001	16.5 ± 10.7	15.3 ± 10.4	19.2 ± 10.7	< 0.0001
Diabetes duration > 10 years, *n* (%)	0 (0.0)	4585 (44.0)	3286 (64.4)	< 0.0001	963 (64.9)	1560 (59.8)	763 (75.4)	< 0.0001
HbA_1c_, %	6.29 ± 0.64	7.45 ± 1.45	7.77 ± 1.59	< 0.0001	7.87 ± 1.70	7.64 ± 1.46	7.98 ± 1.72	< 0.0001
(mmol mol^−1^)	(45.2 ± 7.0)	(57.9 ± 15.8)	(61.4 ± 17.4)		(62.5 ± 18.6)	(60.0 ± 16.0)	(63.7 ± 18.8)	
BMI, kg m^−2^	25.3 ± 2.8	29.0 ± 5.2	29.0 ± 5.0	< 0.0001	29.4 ± 5.2	28.7 ± 4.8	29.3 ± 5.0	< 0.0001
Obesity, *n* (%)	0 (0.0)	3764 (36.1)	1912 (37.5)	< 0.0001	609 (41.1)	902 (34.6)	401 (39.6)	< 0.0001
Waist circumference, cm	95.2 ± 5.8	102.4 ± 10.5	102.8 ± 10.2	< 0.0001	103.4 ± 10.5	102.2 ± 9.9	103.4 ± 10.3	< 0.0001
Triglycerides, mmol l^−1^	1.08 ± 0.58	1.53 ± 0.96	1.68 ± 1.06	< 0.0001	1.82 ± 1.12	1.52 ± 0.93	1.88 ± 1.19	< 0.0001
Total cholesterol, mmol l^−1^	4.01 ± 0.55	4.87 ± 0.96	4.63 ± 1.03	< 0.0001	4.87 ± 1.07	4.51 ± 0.97	4.59 ± 1.05	< 0.0001
HDL cholesterol, mmol l^−1^	1.39 ± 0.39	1.32 ± 0.35	1.23 ± 0.34	< 0.0001	1.24 ± 0.36	1.25 ± 0.33	1.18 ± 0.35	< 0.0001
Non-HDL cholesterol, mmol l^−1^	2.62 ± 0.46	3.55 ± 0.93	3.40 ± 0.98	< 0.0001	3.63 ± 1.03	3.26 ± 0.92	3.42 ± 0.99	< 0.0001
LDL cholesterol, mmol l^−1^	2.13 ± 0.40	2.86 ± 0.83	2.64 ± 0.85	< 0.0001	2.80 ± 0.88	2.58 ± 0.82	2.58 ± 0.86	< 0.0001
Dyslipidemia, *n* (%)	0 (0.0)	8524 (81.7)	4332 (84.9)	< 0.0001	1192 (80.4)	2281 (87.5)	859 (84.9)	< 0.0001
Systolic BP, mmHg	124.7 ± 12.0	137.8 ± 17.4	139.0 ± 19.2	< 0.0001	141.4 ± 19.5	137.1 ± 18.6	140.1 ± 19.8	< 0.0001
Diastolic BP, mmHg	76.9 ± 8.6	79.2 ± 9.2	77.9 ± 9.8	< 0.0001	78.8 ± 10.3	77.7 ± 9.4	76.9 ± 10.0	< 0.0001
Pulse pressure, mmHg	47.9 ± 9.8	58.5 ± 15.2	61.1 ± 16.6	< 0.0001	62.7 ± 17.0	59.4 ± 15.8	63.3 ± 17.4	< 0.0001
Hypertension, *n* (%)	0 (0.0)	7577 (72.7)	4634 (90.8)	< 0.0001	1347 (90.8)	2329 (89.3)	958 (94.7)	< 0.0001
Anti-hyperglycemic treatment, *n* (%)				< 0.0001				< 0.0001
Lifestyle	38 (30.2)	1682 (16.1)	393 (7.7)		101 (6.8)	235 (9.0)	57 (5.6)	
Non-insulin	65 (51.6)	6742 (64.7)	2812 (55.1)		774 (52.2)	1605 (61.5)	433 (42.8)	
Insulin	23 (18.3)	2003 (19.2)	1898 (37.2)		608 (41.0)	768 (29.4)	522 (51.6)	
Lipid-lowering treatment, *n* (%)	0 (0.0)	4149 (39.8)	3089 (60.5)	< 0.0001	680 (45.9)	1760 (67.5)	649 (64.1)	< 0.0001
Anti-hypertensive treatment, *n* (%)	0 (0.0)	6640 (63.7)	4432 (86.9)	< 0.0001	1279 (86.2)	2217 (85.0)	936 (92.5)	< 0.0001
Anti-platelet treatment, *n* (%)	10 (7.9)	2999 (28.8)	3239 (63.5)	< 0.0001	664 (44.8)	1857 (71.2)	718 (70.9)	< 0.0001
Anti-coagulant treatment, *n* (%)	0 (0.0)	258 (2.5)	411 (8.1)	< 0.0001	94 (6.3)	188 (7.2)	129 (12.7)	< 0.0001
Albuminuria, mg day^−1^	10.5 ± 12.1	21.9 ± 35.1	176.8 ± 538.1	< 0.0001	305.1 ± 619.1	27.7 ± 43.5	373.2 ± 880.1	< 0.0001
Serum creatinine, μmol l^−1^	69.7 ± 16.9	72.1 ± 17.39	9.8 ± 50.1	< 0.0001	118.2 ± 66.9	78.9 ± 18.0	126.9 ± 54.0	< 0.0001
eGFR, ml min^−1^·1.73 m^−2^	96.8 ± 15.8	86.2 ± 16.2	67.7 ± 23.8	< 0.0001	56.6 ± 24.3	80.4 ± 15.9	51.6 ± 21.9	< 0.0001
DKD phenotype, *n* (%)				< 0.0001				< 0.0001
No DKD	118 (93.7)	8096 (77.6)	1770 (34.7)		0 (0.0)	1770 (67.9)	0 (0.0)	
Albuminuric DKD with preserved eGFR	7 (5.6)	1690 (16.2)	1269 (24.9)		476 (32.1)	542 (20.8)	251 (24.8)	
Nonalbuminuric DKD	1 (0.8)	641 (6.1)	834 (16.3)		339 (22.9)	296 (11.3)	199 (19.7)	
Albuminuric DKD with reduced eGFR	0 (0.0)	0 (0.0)	1230 (24.1)		668 (45.0)	0 (0.0)	562 (55.5)	
DR, *n* (%)				< 0.0001				< 0.0001
No DR	118 (93.7)	8816 (84.5)	3255 (63.8)		785 (52.9)	1984 (76.1)	486 (48.0)	
Non-advanced DR	6 (4.8)	1138 (10.9)	803 (15.7)		183 (12.3)	453 (17.4)	167 (16.5)	
Advanced DR	2 (1.6)	473 (4.5)	1045 (20.5)		515 (34.7)	171 (6.6)	359 (35.5)	
TOD, *n* (%)	0 (0.0)	0 (0.0)	2495 (48.9)	< 0.0001	1483 (100.0)	0 (0.0)	1012 (100.0)	< 0.0001
ASCVD, *n* (%)								
Any	0 (0.0)	0 (0.0)	2495 (48.9)	< 0.0001	0 (0.0)	2608 (100.0)	1012 (100.0)	< 0.0001
Myocardial infarction	0 (0.0)	0 (0.0)	3620 (70.9)	< 0.0001	0 (0.0)	1281 (49.1)	461 (45.6)	< 0.0001
Coronary revascularization	0 (0.0)	0 (0.0)	1742 (34.1)	< 0.0001	0 (0.0)	1185 (45.4)	394 (38.9)	< 0.0001
Any coronary event	0 (0.0)	0 (0.0)	1579 (30.9)	< 0.0001	0 (0.0)	1774 (68.0)	622 (61.5)	< 0.0001
Stroke	0 (0.0)	0 (0.0)	2396 (47.0)	< 0.0001	0 (0.0)	350 (13.4)	163 (16.1)	< 0.0001
Carotid revascularization	0 (0.0)	0 (0.0)	513 (10.1)	< 0.0001	0 (0.0)	593 (22.7)	263 (26.0)	< 0.0001
Any carotid event	0 (0.0)	0 (0.0)	856 (16.8)	< 0.0001	0 (0.0)	895 (34.3)	397 (39.2)	< 0.0001
Ulcer/gangrene/amputation	0 (0.0)	0 (0.0)	1292 (25.3)	< 0.0001	0 (0.0)	315 (12.1)	241 (23.8)	< 0.0001
Lower limb revascularization	0 (0.0)	0 (0.0)	556 (10.9)	< 0.0001	0 (0.0)	290 (11.1)	160 (15.8)	< 0.0001
Any peripheral event	0 (0.0)	0 (0.0)	450 (8.8)	< 0.0001	0 (0.0)	554 (21.2)	329 (32.5)	< 0.0001
Comorbidities *n* (%)								
Any	22 (17.5)	1668 (16.0)	1097 (21.5)	< 0.0001	327 (22.0)	527 (20.2)	243 (24.0)	< 0.0001
COPD	3 (2.4)	360 (3.5)	311 (6.1)	< 0.0001	94 (6.3)	126 (4.8)	91 (9.0)	< 0.0001
Chronic liver disease	17 (13.5)	811 (7.8)	533 (10.4)	< 0.0001	139 (9.4)	281 (10.8)	113 (11.2)	< 0.0001
Cancer	4 (3.2)	660 (6.3)	367 (7.2)	0.038	127 (8.6)	164 (6.3)	76 (7.5)	0.005

*P* value versus moderate risk of 3-group * and 3-group^†^ comparisons. *ESC* European Society of Cardiology, *HbA*_*1c*_ hemoglobin A_1c_, *BMI* body mass index, *BP* blood pressure, *eGFR* estimated glomerular filtration rate, *DKD* diabetic kidney disease, *DR* diabetic retinopathy, *TOD* target organ damage, *ASCVD* atherosclerotic cardiovascular disease, *COPD* chronic obstructive pulmonary disease

Based on the 2019 classification, only 60 patients (0.4%) were assigned to the moderate-risk category, whereas approximately one third fell in the high-risk category and two thirds fell in the very high-risk category. Of the 63.8% participants assigned to the very high-risk category, 31.8% had ≥ 3 additional ASCVD risk factors; 8.9% had TOD, 3.8% with < 3 and 5.1% with ≥ 3 additional ASCVD risk factors; and 23.1% had previous ASCVD event(s), 8.5% with < 3 and 14.6% with ≥ 3 additional ASCVD risk factors, and 18.0% without and 5.1% with TOD.

Based on the 2021 classification, the number of patients assigned to the moderate-risk category increased but remained negligible (126, 0.8%), whereas the proportion of those falling in the high-risk and very high-risk categories was inverted (approximately two thirds and one third, respectively), compared with the 2019 classification. Of the 32.6% participants assigned to the very high-risk category, 9.5% had TOD only, 16.7% had previous ASCVD only, and 6.5% had both.

Differences between the two classifications (Supplemental Table 2) were due to reallocation of patients with ≥ 3 additional ASCVD risk factors to the high-risk category and a broader definition of TOD, which increased the number of individuals with TOD from 2184 to 2495.

### All-cause mortality according to the 2019 ESC risk categories and subcategories

Crude and sex-adjusted mortality rates increased significantly from the moderate-risk to the very high-risk category; when further adjusting for age, no differences were observed among categories and subcategories (Table 3).

**Table 3:** Mortality rates by 2019 and 2021 ESC risk categories and subcategories

	N	Events	Percent events	Events per 1000 patient-years (95% CI), Unadjusted	*P*	Events per 1000 patient-years (95% CI), adjusted by sex	*P*	Events per 1000 patient-years (95% CI), adjusted by sex & age	*P*
ESC risk categories 2019					< 0.0001		< 0.0001		< 0.0001
Moderate	60	3	5.0	6.20 (2.00–19.21)	Ref	4.42 (1.42–13.79)	Ref	24.38 (7.80–76.21)	Ref
High	5612	710	12.7	16.12 (14.98–17.35)	0.006	11.48 (10.06–13.10)	< 0.0001	9.35 (8.17–10.70)	0.287
Very high	9984	2889	28.9	40.36 (38.92–41.86)	< 0.0001	28.99 (25.89–32.45)	< 0.0001	14.46 (12.81–16.33)	0.483
≥ 3 RFs	4978	1072	21.5	28.67 (27.01–30.44)	< 0.0001	24.17 (21.48–27.18)	< 0.0001	11.60 (10.22–13.18)	0.312
TOD	1386	448	32.3	45.49 (41.47–49.90)	< 0.0001	37.90 (32.88–43.70)	< 0.0001	20.92 (18.06–24.23)	0.663
ASCVD	3620	1369	37.8	56.25 (53.35–59.31)	< 0.0001	46.28 (40.76–52.54)	< 0.0001	21.49 (18.79–24.58)	0.688
ESC risk categories 2021			< 0.0001		< 0.0001		< 0.0001		
Moderate	126	7	5.6	6.87 (3.28–14.40)	Ref	6.08 (2.87–12.87)	Ref	7.50 (3.54–15.88)	Ref
High	10,427	1639	15.7	20.29 (19.34–21.30)	< 0.0001	18.18 (16.21–20.40)	< 0.0001	11.21 (9.93–12.64)	0.194
Very high	5103	1956	38.3	57.00 (54.53–59.58)	< 0.0001	50.67 (44.94–57.13)	< 0.0001	22.47 (19.74–25.57)	< 0.0001
TOD only	1483	587	39.6	58.82 (54.25–63.78)	< 0.0001	51.50 (45.04–58.88)	< 0.0001	22.49 (19.48–25.96)	< 0.0001
ASCVD only	2608	779	29.9	42.17 (39.31–45.24)	< 0.0001	36.55 (31.96–41.81)	< 0.0001	18.15 (15.75–20.91)	< 0.0001
TOD + ASCVD	1012	590	58.3	100.61 (92.81–109.07)	< 0.0001	87.46 (76.16–100.44)	< 0.0001	34.24 (29.52–39.72)	< 0.0001

*ESC* European Society of Cardiology, *CI* confidence interval, *RFs* risk factors, *TOD* target organ damage, *ASCVD* atherosclerotic cardiovascular disease

**Fig. 1:**
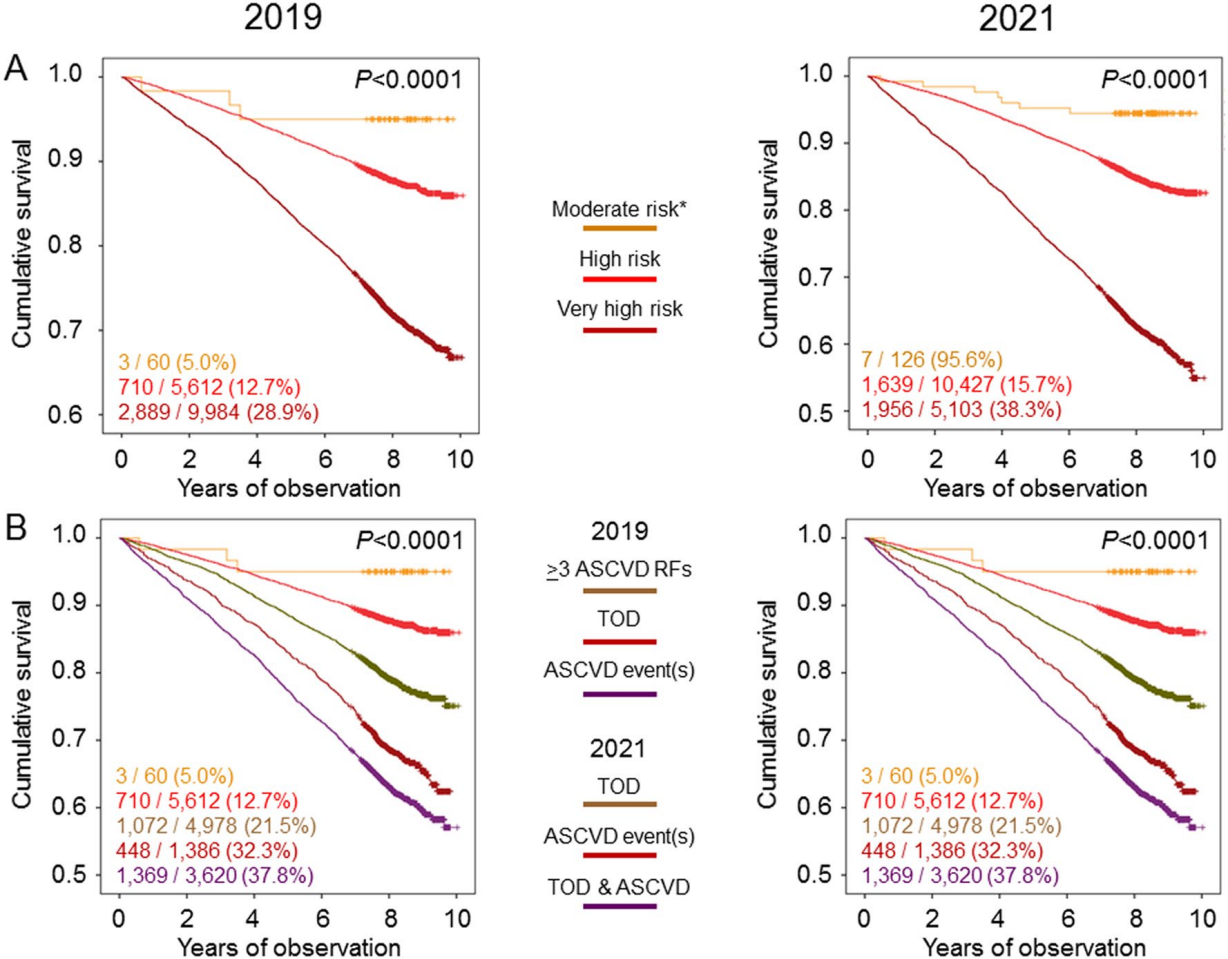
Survival analysis by 2019 and 2021 ESC risk categories. Cumulative survival by Kaplan–Meier analysis according to 2019 and 2021 ESC risk categories (**A**) and subcategories (**B**). Numbers (percentages) of deaths and HRs (95% CI) are shown for each group. *Reference category. ESC = European Society of Cardiology; RFs = risk factors; TOD = target organ damage; ASCVD = atherosclerotic cardiovascular disease

Kaplan–Meier estimates (Fig. 1A) and unadjusted HRs (Table 4) showed the same trend, though mortality risk was not significantly different between the moderate-risk and high-risk categories. The HRs for mortality were not significantly affected when adjusting for sex, comorbidities, and glycemic control, but differences disappeared after further adjustment for additional ASCVD risk factors (Table 4).

**Table 4:** Cox proportional hazards regression, unadjusted and adjusted for sex and severe comorbidities (Model 1), plus HbA_1c_ and anti-hyperglycaemic treatment (Model 2) plus additional ASCVD risk factors (Model 3), according to 2019 and 2021 ESC risk categories and subcategories

	Unadjusted			Model 1			Model 2			Model 3		
	HR	95% CI	*P*	HR	95% CI	*P*	HR	95% CI	*P*	HR	95% CI	*P*
*2019 ESC categories and subcategories*												
Moderate-risk	1.000	–	< 0.0001	1.000	–	< 0.0001	1.000	–	< 0.0001	1.000	–	< 0.0001
High-risk	2.608	(0.839–8.103)	0.098	2.526	(0.813–7.849)	0.109	2.805	(0.903–8.719)	0.075	0.368	(0.117–1.151)	0.086
Very high-risk	6.601	(2.128–20.475)	0.001	6.354	(2.048–19.710)	0.001	6.529	(2.105–20.254)	0.001	0.502	(0.160–1.577)	0.238
Moderate-risk	1.000	-	< 0.0001	1.000	-	< 0.0001	1.000	-	< 0.0001	1.000	-	< 0.0001
High-risk	2.609	(0.839–8.108)	0.097	2.531	(0.814–7.864)	0.109	2.762	(0.888–8.584)	0.079	0.354	(0.113–1.108)	0.074
≥ 3 RFs	4.667	(1.503–14.492)	0.008	4.567	(1.471–14.182)	0.009	4.918	(1.583–15.276)	0.006	0.363	(0.115–1.141)	0.083
TOD	7.466	(2.399–23.237)	0.001	7.243	(2.327–22.542)	0.001	6.672	(2.144–20.766)	0.001	0.565	(0.180–1.778)	0.329
ASCVD	9.279	(2.989–28.804)	< 0.0001	8.645	(2.785–26.837)	< 0.0001	8.501	(2.738–26.394)	< 0.0001	0.620	(0.197–1.949)	0.413
*2021 ESC categories and subcategories*												
Moderate-risk	1.000	–	< 0.0001	1.000	–	< 0.0001	1.000	–	< 0.0001	1.000	–	< 0.0001
High-risk	2.974	(1.415–6.247)	0.004	3.014	(1.435–6.333)	0.004	2.757	(1.311–5.795)	0.007	1.280	(0.606–2.703)	0.518
Very high-risk	8.504	(4.049–17.861)	< 0.0001	8.273	(3.939–17.377)	< 0.0001	6.865	(3.266–14.432)	< 0.0001	2.242	(1.059–4.747)	0.035
Moderate-risk	1.000	–	< 0.0001	1.000	–	< 0.0001	1.000	–	< 0.0001	1.000	–	< 0.0001
High-risk	2.975	(1.416–6.250)	0.004	3.019	(1.437–6.343)	0.004	2.772	(1.319–5.828)	0.007	1.330	(0.630–2.808)	0.455
TOD only	8.797	(4.175–18.535)	< 0.0001	8.572	(4.068–18.060)	< 0.0001	7.108	(3.370–14.991)	< 0.0001	2.240	(1.055–4.759)	0.036
ASCVD only	6.249	(2.969–13.152)	< 0.0001	6.099	(2.898–12.837)	< 0.0001	5.286	(2.510–11.134)	< 0.0001	2.021	(0.953–4.286)	0.066
TOD + ASCVD	15.402	(7.310–32.451)	< 0.0001	14.871	(7.058–31.332)	< 0.0001	11.947	(5.664–25.202)	< 0.0001	3.380	(1.589–7.188)	0.002

*HbA*_*1c*_ hemoglobin A_1c_, *ASCVD* atherosclerotic cardiovascular disease, *ESC* European Society of Cardiology, *RFs* risk factors, *TOD* target organ damage

Within the very high-risk category, death rates (Table 3), Kaplan–Meier estimates (Fig. 1B), and unadjusted and adjusted HRs (Table 4) of patients with TOD were almost equal to those of patients with previous ASCVD event(s), the majority of whom had also TOD and much higher than those of individuals with ≥ 3 additional ASCVD risk factors.

### All-cause mortality according to the 2021 ESC risk categories and subcategories

Crude and sex-adjusted mortality rates increased significantly from the moderate-risk to the very high-risk category; when further adjusting for age, differences persisted only between the high-risk and very high-risk categories (Table 3).

Kaplan–Meier estimates (Fig. 1C) and unadjusted HRs (Table 4) showed the same trend. The HRs for mortality were not significantly affected when adjusting for sex, comorbidities, and glycemic control, but risk remained significantly higher only in the very high-risk versus the moderate-risk category when accounting for additional ASCVD risk factors mortality (Table 4).

Within the very high-risk category, death rates (Table 3), Kaplan–Meier estimates (Fig. 1D), and unadjusted and adjusted HRs (Table 4) were much higher in the subcategories with previous ASCVD event(s) and TOD and, to a lesser extent, TOD only than in the subcategory with previous ASCVD event(s) only. Moreover, when further adjusting for additional ASCVD risk factors, the HRs for mortality remained significantly higher versus the moderate-risk category in patients with TOD only or previous ASCVD event(s) and TOD (Table 4).

## Discussion

This analysis of patients with type 2 diabetes from the RIACE cohort showed that very few of these individuals fell in the moderate-risk category according to both ESC classifications. Moreover, the proportion of participants in the very high-risk risk category was approximately twice higher than that of participants in the high-risk category according to the 2019 classification. These findings are consistent with previous reports assessing the distribution of patients from diabetes outpatient clinics according to the 2019 ESC classification [19, 20], in which the proportion of individuals at moderate risk was similar and that of those at very high risk was even higher than in the RIACE cohort. However, using the 2021 classification, the proportion of participants in the very high-risk category became about half of that of participants in the high-risk category, due to the reallocation of patients with at least three additional ASCVD risk factors to the high-risk category. The finding that these patients showed a much lower mortality risk than those with TOD and/or previous ASCVD event(s) and closer to those in the high-risk category as in the 2019 classification is consistent with the revised risk stratification system proposed in the 2021 guidelines. Finally, within the very high-risk category, the mortality risk of individuals with TOD only was almost similar to that of patients with previous ASCVD event(s), with and without TOD, according to the 2019 classification, and higher than that of patients with previous ASCVD event(s) only, according to the 2021 classification. These findings might reflect the strong, independent association of microangiopathy, especially DKD, with measures of macroangiopathy such as left ventricular hypertrophy [21] and coronary calcification [22]. It is in fact plausible that, in individuals with TOD due to DKD and/or DR but without established ASCVD, the overall risk of death results from both overt microvascular and subclinical macrovascular diseases, thus indicating the need for noninvasive assessment of subclinical ASCVD and treatment with drugs providing protection from both ASCVD and DKD [23].

Taken together, the results of this analysis provide important insights into the risk stratification of patients with type 2 diabetes, though comparison between the ESC classification system and the existing prediction tools is not feasible as the former allows only a broad categorization of patients according to the risk of death from ASCVD, instead of quantifying the predicted risk. As stated above, prediction algorithms have several pitfalls that limit their performance in these individuals [8]. One limitation is that many of them have been derived from general population samples and not established (or validated) in people with type 2 diabetes. The ESC guidelines do in fact discourage to apply those from the general population to patients with diabetes [11, 12], though comparisons with diabetes-specific algorithms have not univocally shown that the latter ones perform better [8, 24]. Another limitation is the time-period when they were developed, as some of them date back to several years ago and, hence, do not consider the impact of recent therapeutic advances on ASCVD risk. Moreover, they estimate risk of different outcomes, including all-cause or ASCVD mortality and ASCVD events, with most of them being specific for myocardial infarction and stroke without considering other events, such as heart failure and peripheral artery disease. More importantly, they are mainly based on ASCVD risk factors, as only few of them consider previous ASCVD events [25, 26], thus being applicable also to patients with established ASCVD, and/or the presence of TOD, which was found to be associated with an extremely elevated mortality risk among the RIACE participants. In fact, many of the most used algorithms, either derived from the general population [9, 27–30] or people with type 2 diabetes [31, 32], do not include measures of TOD. In addition, the remaining algorithms consider only measure(s) of kidney damage, i.e., serum creatinine or eGFR [26, 33], albuminuria [34], or both [25, 35]. Therefore, it is not surprising that the Risk Equations for Complications of Type 2 Diabetes (RECODe), a tool for predicting complications and death that includes previous ASCVD events, serum creatinine, and albumin:creatinine ratio, was found to perform better than six of the above algorithms [25].

In this regard, the ESC classification system appears to be superior as it considers also TODs other than DKD, such as retinopathy and neuropathy. However, other complications are not taken into account, such as non-alcoholic fatty liver disease, which has been shown to independently predict fatal and non-fatal ASCVD events [36], and particularly measures of subclinical atherosclerosis. These measures include (a) coronary, carotid, or lower limb artery stenosis, as assessed by computed tomography angiography or ultrasound, which the American Diabetes Association guidelines consider an index of high risk if higher than 50% [37]; (b) functional imaging; (c) ankle–brachial index; and (d) coronary artery calcium scoring. All these are considered as risk modifiers, particularly the latter [12], which is recommended for coronary risk assessment in asymptomatic adults at intermediate 10-year risk (10% to 20%) or low-to-intermediate 10-year risk (6% to 10%), with calcium score driving reclassification of these individuals to the low-risk or high-risk category [38].

However, the ESC stratification systems do not appear to reflect the wide range of ASCVD risk observed in people with type 2 diabetes [39], as attested by the negligible number of patients in the moderate-risk category with both ESC classifications and the assignment of the majority of participants to the very high-risk category with the 2019 classification. This is likely because they do not take into account the degree of ASCVD risk factor control, at variance with all the existing risk prediction tools. In fact, optimal treatment of ASCVD risk factors in individuals with type 2 diabetes was found to significantly reduce or even eliminate the excess risk of death and ASCVD events compared to non-diabetic controls [40, 41]. As a consequence, the ESC stratification systems might overestimate mortality risk in a great number of people with type 2 diabetes and, as acknowledged by the ESC Scientific Document Group [12], may not be appropriate to accurately quantify risk differences. This interpretation is in keeping with a systematic review and network meta-analysis of randomized controlled trials [42] and a clinical practice guideline based on it [43], which stratified adults with type 2 diabetes into five risk categories using the RECODe prediction model [44] and assigned those with ≤ 3 and > 3 additional ASCVD risk factors to the very low- and low-risk category, respectively.

Strength of our study includes the large sample size, the assessment of a wide range of clinical parameters, and the completeness of baseline and follow-up data. However, there are several limitations. First, the lack of information on the causes of death did not allow detecting ASCVD deaths, to which the ESC classification systems specifically refer. Second, results may have been affected by the lack of information on left ventricular hypertrophy and diabetic neuropathy, which were considered for defining TOD in the 2019 and 2021 classification, respectively [11, 12]. Third, the study findings may not be applicable to the general ambulatory population, as only part of the individuals with type 2 diabetes attend Diabetes Clinics in Italy. Finally, the observational design makes causal interpretation impossible.

In conclusion, risk stratification of patients with type 2 diabetes from the RIACE cohort showed that only a few of them fell in the moderate-risk category according to both ESC classifications and that the majority of participants were assigned to the very high-risk category according to 2019 classification, due to the inclusion of those with at least three additional ASCVD risk factors. This suggests that the ESC stratification systems might overestimate mortality risk in patients with type 2 diabetes without TOD and ASCVD because they do not take into account the degree of ASCVD risk factor control. Reallocating individuals with at least three additional ASCVD risk factors to the high-risk category as in the 2021 classification was consistent with the observed all-cause mortality data. Mortality risk increased across categories according to both classifications, but differences among categories were more evident using the 2021 stratification criteria. Within the very high-risk category, risk of death was found to be particularly high in the presence TOD (namely microangiopathy), irrespective of established ASCVD, possibly due to the association with subclinical vascular disease.

## Supplementary Information

Below is the link to the electronic supplementary material.

Supplementary file1 (DOC 65 KB)

## Data Availability

The datasets used and/or analyzed during the current study are available from the corresponding author on reasonable request.

## Abbreviations

ASCVD: Atherosclerotic cardiovascular disease
ESC: European Society of Cardiology
TOD: Target organ damage
RIACE: Renal Insufficiency And Cardiovascular Events
eGFR: Estimated glomerular filtration rate
BP: Blood pressure
BMI: Body mass index
HbA_1c_: Hemoglobin A_1c_
DKD: Diabetic kidney disease
DR: Diabetic retinopathy
Ci: Confidence interval
HR: Hazard ratio
RECODe: Risk Equations for Complications of Type 2 Diabetes
