# Supplementary material for: Risk of all-cause mortality according to the European Society of Cardiology risk categories in individuals with type 2 diabetes: the Renal Insufficiency And Cardiovascular Events (RIACE) Italian Multicenter Study
Source: Acta Diabetol. 2022 Jul 28;59(10):1369–81. doi: 10.1007/s00592-022-01942-8 (PMC9402482; doi:10.1007/s00592-022-01942-8)
Supplement: Supplementary file 1 — Supplementary file1 (DOC 65 KB) [file 592_2022_1942_MOESM1_ESM.doc]

**The RIACE Study Group.**

**The RIACE Steering Committee**

Giuseppe Pugliese (Coordinator), Giuseppe Penno (Secretary), Anna Solini, Enzo Bonora, Emanuela Orsi, Roberto Trevisan, Luigi Laviola, Antonio Nicolucci.

**Participating Diabetes Centres**

1. Azienda Ospedaliera Sant'Andrea, Roma (Coordinating Centre): Giuseppe Pugliese, Lucilla Bollanti, Elena Alessi, Martina Vitale, and Tiziana Cirrito.
2. Ospedale Le Molinette, Torino: Paolo Cavallo-Perin, Gabriella Gruden, and Bartolomeo Lorenzati.
3. Ospedale San Luigi Gonzaga, Orbassano: Franco Cavalot, Mariella Trovati, Leonardo Di Martino, and Fabio Mazzaglia.
4. Ospedale San Raffaele, Milano: Giampaolo Zerbini, Valentina Martina, Silvia Maestroni, and Valentina Capuano.
5. IRCCS “Cà Granda – Ospedale Maggiore Policlinico”, Milano: Emanuela Orsi, Eva Palmieri, Elena Lunati, Valeria Grancini, and Veronica Resi.
6. Ospedale San Paolo, Milano: Antonio Pontiroli, Annamaria Veronelli, and Barbara Zecchini.
7. Ospedale San Giuseppe, Milano: Maura Arosio, Laura Montefusco, Antonio Rossi, and Guido Adda.
8. ASST - Ospedale Papa Giovanni XXIII, Bergamo: Roberto Trevisan, Anna Corsi, and Mascia Albizzi.
9. Ospedale Maggiore, Verona: Enzo Bonora, and Giacomo Zoppini.
10. Policlinico Universitario, Padova: Angelo Avogaro, and Monica Vedovato.
11. Ospedale Cisanello, Azienda Ospedaliero-Universitaria Pisana, Pisa: Giuseppe Penno, Laura Pucci, Daniela Lucchesi, Eleonora Russo, and Monia Garofolo.
12. Ospedale Santa Chiara, Azienda Ospedaliero-Universitaria Pisana, Pisa: Anna Solini.
13. Ospedale Le Scotte, Siena: Francesco Dotta, Cecilia Fondelli, and Laura Nigi.
14. Policlinico Umberto I, Roma: Susanna Morano, Tiziana Filardi, Irene Turinese, and Marco Rossetti.
15. Ospedale S. Maria Goretti, Latina: Raffaella Buzzetti and Chiara Foffi.
16. Ospedali Riuniti, Foggia: Mauro Cignarelli, Olga Lamacchia, Sabina Pinnelli, and Lucia Monaco.
17. Policlinico Universitario, Bari: Francesco Giorgino, Luigi Laviola, and Annalisa Natalicchio.
18. Policlinico Mater Domini, Catanzaro: Giorgio Sesti and Francesco Andreozzi.
19. Policlinico Monserrato, Cagliari: Marco Giorgio Baroni, Giuseppina Frau, and Alessandra Boi.

**Supplemental Table 1.** ESC risk categories according to the 2019 and 2021 classification, as originally defined and modified for the purpose of this analysis**.**

| **Risk category** | **Original 2019 criteria** | **Modified 2019 criteria** | **Original 2021 criteria** | **Modified 2021 criteria** |
| --- | --- | --- | --- | --- |
| Moderate risk | Young patients (aged <50 years) with short-standing DM (<10 years), no additional ASCVD risk factors, and no evidence of established ASCVD or severe TOD | Young patients (aged <50 years) with short-standing DM (<10 years), no additional ASCVD risk factors*, and no evidence of established ASCVD or severe TOD | Patients with well controlled short-standing (<10 years) DM, no additional ASCVD risk factors, and no evidence of established ASCVD and severe TOD | Patients with well controlled (HbA_1c_ <7%) short-standing (<10 years) DM, no additional ASCVD risk factors*, and no evidence of established ASCVD and severe TOD |
| High risk | Patients with short-standing DM (>10 years) and/or 1-2 additional ASCVD risk factors, and no evidence of established ASCVD or severe TOD | Patients with older age (>50 years) and/or long-standing DM (>10 years) and/or 1-2 additional ASCVD risk factors*, and no evidence of established ASCVD and severe TOD | Patients with DM without established ASCVD and/or severe TOD and not fulfilling the moderate risk criteria | Patients with not well controlled (>7%) and/or long-standing (>10 years) DM and/or any additional ASCVD risk factor*, and no evidence of established ASCVD and severe TOD |
| Very high risk | Patients with DM and >3 additional ASCVD risk factors and/or established ASCVD and/or severe TOD, i.e.;   - proteinuria - eGFR <30 mL/min/1.73 m^2^ - left ventricular hypertrophy - retinopathy | Patients with DM and >3 additional ASCVD risk factors* and/or established ASCVD and/or severe TOD, i.e.;   - proteinuria - eGFR <30 mL/min/1.73 m^2^ - advanced retinopathy | Patients with DM and established ASCVD and/or severe TOD, i.e.:   - eGFR <45 mL/min/1.73 m^2^ irrespective of albuminuria - eGFR 45-59 mL/min/1.73 m^2^ and microalbuminuria (ACR 30 -300 mg/g) - proteinuria (ACR >300 mg/g) - presence of microvascular disease (microalbuminuria plus retinopathy plus neuropathy) | Patients with DM and established ASCVD and/or severe TOD, i.e.:   - eGFR <45 mL/min/1.73 m^2^ irrespective of albuminuria - eGFR 45-59 mL/min/1.73 m^2^ and microalbuminuria (ACR 30 -300 mg/g) - proteinuria (ACR >300 mg/g) - presence of microvascular disease (eGFR 45-59 mL/min/1.73 m^2^ and/or microalbuminuria plus advanced retinopathy) |

* Additional ASCVD risk factors:

- Age (>70 years)
- Smoking (current)
- Obesity (BMI >30)
- Dyslipideamia (total cholesterol >5.18 mmol/l and/or LDL cholesterol >2.59 mmol/l and/or triglycerides >1.70 mmol/l and/or lipid-lowering treatment)
- Hypertension (systolic BP ≥140 mmHg and/or diastolic BP ≥90 and/or anti-hypertensive treatment)

ESC = European Society of Cardiology; DM = diabetes mellitus; ASCVD = cardiovascular disease; TOD = target organ damage; eGFR = estimated glomerular filtration rate; ACR = albumin:creatinine ratio; HbA_1c_ = hemoglobin A_1c_; BMI = body mass index; BP = blood pressure.

**Supplemental Table 2.** Cross-classification of patients according to the 2019 and 2021 guidelines.

|  | | **2021 risk categories** | | | |
| --- | --- | --- | --- | --- | --- |
|  |  | **Moderate-risk** | **High-risk** | **Very high-risk** | **Total** |
| **2019 risk categories** | **Moderate-risk** | 35 | 25 | 0 | 60 |
|  | **High-risk** | 89 | 5,338 | 185 | 5,612 |
|  | **Very high-risk** | 2 | 5,064 | 4,918 | 9,984 |
|  | **Total** | 126 | 10,427 | 5,103 | 15,656 |

ESC = European Society of Cardiology.
